# Chia Seed Does Not Improve Cognitive Impairment in SAMP8 Mice Fed with High Fat Diet

**DOI:** 10.3390/nu10081084

**Published:** 2018-08-14

**Authors:** Yehua Rui, Menglian Lv, Jie Chang, Jiaying Xu, Liqiang Qin, Zhongxiao Wan

**Affiliations:** 1 Department of Nutrition and Food Hygiene, School of Public Health, Soochow University, Suzhou 215123, China; Ruiyehua2018@163.com (Y.R.); lvmenglian23@163.com (M.L.); qinliqiang@suda.edu.cn (L.Q.); 2 Department of Occupational and Environmental Health, School of Public Health, Soochow University, 199 Ren’ai Road, Suzhou 215123, China; jchang@suda.edu.cn; 3 School of Radiation Medicine and Protection, Soochow University, 199 Ren’ai Road, Suzhou 215123, China; xujiaying@suda.edu.cn; 4 Jiangsu Key Laboratory of Preventive and Translational Medicine for Geriatric Disease, Soochow University, 199 Ren’ai Road, Suzhou 215123, China

**Keywords:** chia seed, Alzheimer’s disease, amyloid β, Tau, neuro-inflammation

## Abstract

Background: Chia seed is an ancient seed with the richest plant source of α-linolenic acid, which has been demonstrated to improve metabolic syndrome associated risk factors. Under high fat diet (HFD) condition, the senescence-accelerated mouse-prone 8 (SAMP8) mice demonstrated worsen Alzheimer’s disease (AD) related pathology compared to low fat diet fed SAMP8 mice. Objective: To explore whether chia seed supplementation might improve cognitive impairment under aging and metabolic stress via high fat diet (HFD) fed SAMP8 mice as a model. Design: SAMP8 mice and senescence-accelerated mouse-resistant 1 (SAMR1) were randomized into 4 groups, i.e., SAMR1 low fat diet group (SAMR1-LFD), SAMP8-HFD and SAMP8-HFD group supplemented with 10% chia seed (SAMP8-HFD+Chia). At the end of the intervention, cognitive function was measured via Morris water maze (MWM) test. Hippocampus and parietal cortex were dissected for further analysis to measure key markers involved AD pathology including Aβ, tau and neuro-inflammation. Results: During navigation trials of MWM test, mice in SAMP8-LFD group demonstrated impaired learning ability compared to SAMR1-LFD group, and chia seed had no effect on learning and memory ability for HFD fed SAMP8 mice. As for Alzheimer’s disease (AD) related pathology, chia seed not only increased α-secretase such as ADAM10 and insulin degrading enzyme (IDE), but also increased β-secretase including beta-secretase 1 (BACE1) and cathepsin B, with an overall effects of elevation in the hippocampal Aβ_42_ level; chia seed slightly reduced p-Tauser404 in the hippocampus; while an elevation in neuro-inflammation with the activation of glial fibrillary acidic protein (GFAP) and Ibα-1 were observed post chia seed supplementation. Conclusions: Chia seed supplementation did not improve cognitive impairment via MWM in HFD fed SAMP8 mice. This might be associated with that chia seed increased key enzymes involved both in non-amyloidogenic and amyloidogenic pathways, and neuro-inflammation. Future studies are necessary to confirm our present study.

## 1. Introduction

Alzheimer’s disease (AD), as a progressive neurodegenerative disorder, is the main cause of dementia for the elderly. The exact causes of AD remain elusive although it is tied to factors such as genes, environment, and lifestyle. It has now been well established that obesity [1], insulin resistance [2], and dyslipidemia [3] are independent risk factors for AD. Therefore, strategies that could improve obesity, insulin resistance and dyslipidemia might be efficacious in the prevention and treatment of AD.

Chia (*Salvia hispanica* L.) is an ancient seed that belongs to the Lamiaceae family, which is native to southern Mexico and northern Guatemala [4]. Chia seeds contain the richest botanical source of [α-linolenic acid (ALA) 18:3, *n*-3], as well as a high amount of fibers, minerals, polyphenolic compounds mainly including quercetin, myricetin and cholorogenic acid and a good source of protein [5]. Over the past two decades, evidence from both human [6,7,8,9] and animal studies [10,11] have suggested that chia seed is beneficial for improving glucose tolerance and insulin resistance [6,7,8,10], adiposity [11] and disordered lipid profiles [9,11]. Therefore, chia seed might be a promising measure in the prevention and treatment of AD via improving AD related risk factors such as insulin resistance, obesity and dyslipidemia.

In addition, Poudyal et al. [12] reported that 8 weeks 5% chia seed supplementation in high fat diet rats increased *n*-3 : *n*-6 ratio in plasma and multiple tissues including heart, liver and retroperitoneal adipose tissue. In human, supplementation of chia seeds have been reported to increase plasma ALA in postmenopausal women [13], individuals with type 2 diabetes [14], and overweight adults [15], although the effects on EPA remain inconsistent with both elevation [13,14] and no alteration [15] have been reported. Increasing evidence began to support that ALA supplementation, *per se*, could improve AD associated pathology and cognitive impairment. For example, Gao et al. [16] reported that long-term ALA supplementation (12 months) in aged rats prevented age related cognitive dysfunction and neuropathology via suppression of PERK/eIF2α signaling pathway. Lee et al. [17] reported that ALA-rich perilla oil improved Aβ_25–35_ induced learning and memory dysfunction in mice. This further suggests that chia seed might be a promising candidate for the prevention or treatment of AD considering it has the richest plant source of ALA. Whereas, scanty data are available regarding how chia seed supplementation will affect AD related cognitive impairment, and what might be the potential mechanisms involved in.

The senescence-accelerated mouse-prone 8 (SAMP8) mice demonstrate many features normally occur in the pathogenesis of AD such as abnormal APP processing by β and γ secretases and altered amyloid β (Aβ) proteins [18], elevated phosphorylation of tau [19], as well as synaptic and dendritic alterations [20]. Therefore, SAMP8 mice have been widely used as a suitable model for researching aging associated neurodegenerative diseases including AD. Of interest, Mehla et al. [21] reported that under high fat diet (HFD) condition, SAMP8 mice would demonstrate worsen AD related pathology compared to low fat diet fed SAMP8 mice, which indicates that HFD induced SAMP8 mice may represent the metabolic model of AD. Collectively, the aim of this study is to explore whether chia seed, a rich source of ALA might improve cognitive impairment under aging and metabolic stress via HFD fed SAMP8 mice as a model, as well as the potential mechanisms involved in.

## 2. Materials and Methods

### 2.1. Materials

Reagents for SDS-PAGE were from Beyotime Institute of Technology (Haimen, Jiangsu, China). Molecular weight marker and nitrocellulose membranes for SDS-PAGE were from Bio-Rad (Berkeley, CA, USA). Immobilon western chemiluminescent HRP substrate (cat#WBKLS0100) was purchased from Millipore (Bedford, MA, USA). Antibodies against amyloid precursor protein (APP) (cat#2450), beta-secretase 1 (BACE1) (cat# 5606), tau (cat#4019), glial fibrillary acidic protein (GFAP) (cat#3670), p25/35 (cat#2680), PP2A (cat#2041) and beta-Actin (cat#4970) were purchased from Cell Signaling (Danvers, MA, USA). Antibodies against p-Tau serine396 (cat#YP0263), p-Tau serine404 (cat#YP0264) and cyclin-dependent kinases 5 (CDK5) (cat#YT0835) were from ImmunoWay Biotechnology Company (Plano, TX, USA). Antibodies against insulin degrading enzyme (IDE) (cat#ab32216) and cathepsin B (cat#ab58802) were from Abcam (Shanghai, China). An antibody against Aβ_42_ (cat#805501) was purchased from Biolegend (San Diego, CA, USA). An antibody against ADAM10 (cat#sc-48400) was from Santa Cruz (Dallas, Texas, USA). Horseradish peroxidase-conjugated donkey anti-rabbit and goat anti-mouse IgG secondary antibodies were purchased from Jackson Immuno-Research Laboratories (West Grove, PA, USA). Chia seed was obtained from NOW FOODS with a purity of ≥98% (Bloomingdale, IL, USA). The fatty acid composition of chia seed is shown in Table 1.

### 2.2. Treatment of Animals

Ten week old male senescence-accelerated mouse resistant 1 (SAMR1) (*N* = 8) and SAMP8 mice (*N* = 24) were purchased from Animal Service of Health Science Center, Peking University (Beijing, China). All animals were treated in accordance with the Guidelines in the Care and Use of Animals and was approved by the Animal Studies Committee of Soochow University, Suzhou, China. Mice were housed together with two animals of each group in standard plastic mouse cages (30 × 20 × 13 cm^3^) and were acclimated to the animal housing facility for 1 week before the intervention. At 12 weeks of age, mice were randomly assigned into 4 groups of 8 animals each, i.e., SAMR1 low fat diet group (SAMR1-LFD), SAMP8 low fat diet group (SAMP8-LFD), SAMP8 high fat diet group (SAMP8-HFD), SAMP8 high fat diet combined with chia seed intervention group (SAMP8-HFD+Chia). For the SAMR1-LFD and SAMP8-LFD group, the mice were allowed a low fat diet *ad libitum* during the whole period of experimentation (i.e., 18 weeks). The low fat diet was purchased from Research diets INC (cat#D12450J, New Brunswick, NJ, USA) and contained fat 10% Kcal, carbohydrate 70% Kcal and protein 20% Kcal. For the SAMP8-HFD group, the mice were allowed a high fat diet *ad libitum* during the whole period of experimentation (i.e., 18 weeks). The high fat diet contained fat 60% Kcal, carbohydrate 20% Kcal and protein 20% Kcal, and was purchased from Research diets INC. (cat# D12492). For the SAMP8-HFD+Chia group, the mice were allowed an HFD *ad libitum* supplemented with 10% chia seed (i.e., 10 g chia seed powder dissolved in 100 g HFD during the whole period of experimentation). The high fat diet was the same as above and chia seed powder was added into the food following the recipe strictly by the researchers. We chose these dosages of chia seed were mainly based on published findings from others [10,12]. Body weight and food intake were determined every other week during the study. As for the measurement of food intake, every other week, we first weighed the food and then provided the weighed food to the mice, ~48 h later, we weighed the leftover food, and food intake was evaluated via the difference value.

### 2.3. Glucose and Insulin Tolerance Tests

Two days post-the last bout of chia seed intervention, intraperitoneal glucose (GTT) and insulin tolerance tests (ITT) were performed. Mice were fasted for 6 h prior to an I.P. injection of glucose (1.5 g/kg body weight) during the GTT. About 48 h later, mice were given an I.P. injection of insulin (0.5 IU/kg body weight) with free access to food for ITT. Blood glucose levels were determined by tail vein sampling via a glucometer at the following intervals (i.e., 0, 15, 30, 45, 60, 90 and 120 min). Changes in glucose over time were plotted, and the area under the curve (AUC) was calculated for each using the trapezoidal method.

### 2.4. Morris Water Maze (MWM) Test

Two days post-ITT, Morris water maze test was performed to evaluate hippocampal dependent, spatial learning and memory retention ability of mice [22]. In brief, the maze was a round tank (120 cm in diameter and 30 cm in height) filled with water (24 ± 1 °C). The tank was divided into 4 quadrants, one of which contained a hidden circular escape platform (10 cm in diameter, 24 cm in height, and 1.0 cm below the surface of the water.) On 4 consecutive days, oriented navigation trials were performed 4 times per day, between 9:00 a.m. and 2:00 p.m. In each trial, the animal was placed into the water from a different quadrant and had 60 s to track for the platform. If the mouse swam successfully onto the platform within 60 s, it was allowed to rest on the platform for 10 s. If the mouse failed to find the platform within the given time, it was guided to the platform to stay for 20 s. The time that a mouse took to reach the submerged platform (escape latency) was recorded to assess spatial learning ability. On the fifth day, a spatial probe trial was performed to assess the memory retention ability of mice with the platform removed. Mice were placed in the tank in the quadrant opposite to the quadrant that previously contained the platform, and had 60 s to swim freely. The time spent by a mouse in the target quadrant in which the platform was hidden, and the number of mice exactly crossing over the previous position of the platform were recorded. Ethovision XT tracking software (Leesburg, VA, USA) was used for data collection and analysis.

### 2.5. Brain Tissue Collection and Preservation

Two days after the behavioral test, the mice were euthanized, and their brains were immediately analyzed. Then the hippocampus and parietal cortex from the hemi-brains were dissected, snap frozen in liquid nitrogen and stored at −80 °C for further Western blot analysis. The other hemispheres were fixed in 4% paraformaldehyde for a week before 30 μm coronal brain sections embedded in paraffin were obtained for immunofluorescence assay. 

### 2.6. Western Blot Analysis

Proteins were extracted from the hippocampus and parietal cortex of the mice, and the content of APP, BACE1, cathepsin B, ADAM10, IDE, Aβ_42_, p-Tau ser396 and ser404, CDK5, p25/35, PP2A and GFAP were determined by Western blotting as described previously by our laboratory [23,24,25]. Immobilon western chemiluminescent HRP substrate were used for the visualization of signals and signals were captured through a Syngene chemi-imaging system (Waltham, MA, USA). Subsequently bands were quantified by densitometry via Gene Tool according to the manufacturer’s instructions (SynGene, ChemiGenius2, Perkin Elmer, Waltham, MA, USA). Protein contents of phosphorylated protein were quantified and normalized to the total levels of these proteins. Beta actin was considered as internal controls.

### 2.7. Aβ_42_ and Microglia Immune-Staining

Brain paraffin sections were first dehydrated with 70% ethanol for 15 min, then put in citric acid (pH 6.0) for antigen retrieval, thereafter blocked with 3% bovine serum albumin in PBS. Next, sections were incubated with primary antibodies (Aβ_42_, 1/500, cat#805501, Biolegend; Ibα-1,1/500, cat#016-26461, Wako, Osaka, Japan) in 0.3% Triton X-100 in PBS with 5% blocking serum overnight at 4 °C. Thereafter, the following secondary antibodies (Streptavidin Alexa Fluor 555 conjugate, 1/1000, S32355, Invitrogen, Carlsbad, CA, USA for Aβ_42_; Alexa Fluor 488 goat anti-rabbit, 1/500, A-11008, Invitrogen, for Ibα-1) were used for incubation for 1 h at RT in the dark, sections were then re-stained with DAPI (cat#D1306, Invitrogen) for 10 min and mounted on slides. Images were scanned using Pannoramic Midi (3D Histech Ltd., Budapest, Hungary).

### 2.8. Statistical Analysis

Data are expressed as means ± SEM. For the MWM test, escape latency times in the hidden platform trial were analyzed via two-way ANOVA of repeated measures, while one-way ANOVA was conducted for the probe trial followed with Tukey’s post hoc test, as well as for all biochemical data. SPSS version 16.0 statistical analysis package (SPSS Inc., Chicago, IL, USA) were utilized for the analyses. Statistical significance was established at a *p* < 0.05.

## 3. Results

### 3.1. Body Weight

**Figure 1:**
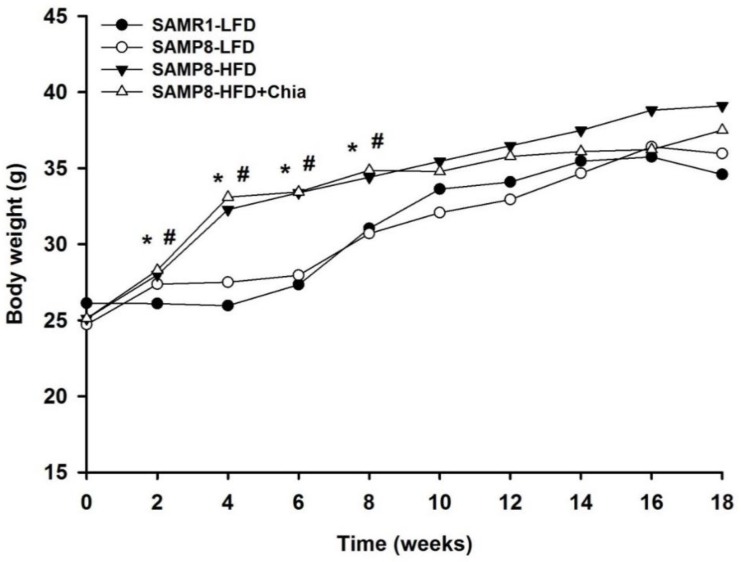
Body weight of mice measured every other week. * *p* < 0.05 versus SAMR1-LFD group within the same day; # *p* < 0.05 versus SAMP8-LFD group within the same day.

As shown in Figure 1, compared to SAMR1-LFD group, there is no difference for initial body weight, while the body weight from both SAMP8-HFD and SAMP8-HFD+Chia group at week 2, 4, 6 and 8 is significantly higher compared to both SAMR1-LFD and SAMP8-LFD group, thereafter there is no difference for body weight among groups. The body weight of mice from SAMR1-LFD, SAMP8-LFD, SAMP8-HFD and SAMP8-HFD+Chia group at 2, 4, 6 and 8 weeks were (26.1 ± 0.5, 27.4 ± 0.6, 28.0 ± 0.5, 28.3 ± 0.5), (26.0 ± 0.4, 27.5 ± 0.5, 32.3 ± 0.8, 33.1 ± 0.8), (27.3 ± 0.4, 28.0 ± 0.4, 33.4 ± 0.9, 33.4 ± 0.7) and (31.0 ± 0.6, 30.7 ± 0.7, 34.4 ± 1.3, 34.9 ± 1.1) g, respectively. 

### 3.2. Glucose and Insulin Tolerance Test

**Figure 2:**
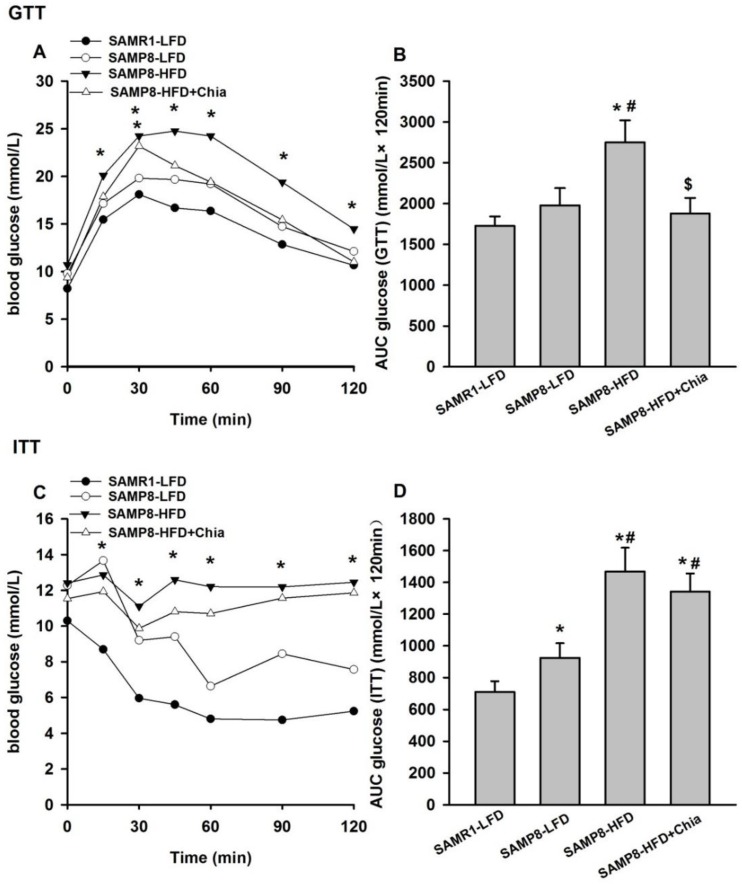
Intraperitoneal glucose (GTT) and insulin tolerance tests (ITT) of mice from different groups. Glucose levels (**A**) at different timepoints and total area under the curve (AUC); (**B**) post-glucose injection. Glucose levels (**C**) at different timepoints; and AUC (**D**) post-insulin injection. Data are presented as means + SEM for 8 mice per group.* *p* < 0.05 versus SAMR1-LFD group within the same timepoint in (**A**,**C**). * *p* < 0.05 versus SAMR1-LFD group in (**B**,**D**); # *p* < 0.05 versus SAMP8-LFD group in (**B**,**D**); $ *p* < 0.05 versus SAMP8-HFD group in **(B**).

During GTT, compared to SAMR1-LFD mice, SAMP8-HFD group mice have elevated glucose level at 15, 30, 45, 60, 90 and 120 min, and SAMP8-HFD+Chia group mice have elevated glucose level only at 30 min (Figure 2A); compared to both SAMR1-LFD and SAMP8-LFD group, the total AUC for GTT is higher in SAMP8-HFD group, and compared to SAMP8-HFD group, SAMP8-HFD+Chia group has significantly reduced total AUC (Figure 2B). The total AUC of GTT from SAMR1-LFD, SAMP8-LFD, SAMP8-HFD and SAMP8-HFD+Chia group were 1728.94 ± 113.49, 1978.71 ± 210.7, 2749.75 ± 270.86, 1878.75 ± 189.53 mmol/L × 120 min, respectively. During ITT, compared to SAMR1-LFD group, SAMP8-LFD, SAMP8-HFD and SAMP8-HFD+Chia group have elevated glucose level at 15, 30, 45, 60, 90 and 120 min (Figure 2C); compared to SAMR1-LFD group, SAMP8-LFD, SAMP8-HFD and SAMP8-HFD+Chia group also have increased total AUC, and the total AUC in SAMP8-HFD and SAMP8-HFD+Chia group are also significantly higher compared to SAMP8-LFD group (Figure 2D). The total AUC of ITT from SAMR1-LFD, SAMP8-LFD, SAMP8-HFD and SAMP8-HFD+Chia group were 710.06 ± 66.91, 924.56 ± 91.97, 1468.22 ± 149.77, 1342.29 ± 113.46 mmol/L × 120 min, respectively. Collectively, it is indicated that HFD resulted in impaired glucose tolerance in SAMP8 mice, and chia seed is able to reverse HFD induced glucose intolerance; SAMP8 mice at low fat diet has impaired insulin tolerance test compared to SAMR1-LFD group; HFD in SAMP8 mice further worsen the insulin tolerance, while chia seed is unable to reverse HFD induced insulin intolerance in SAMP8 mice.

### 3.3. Cognitive Function via MWM Test

**Figure 3:**
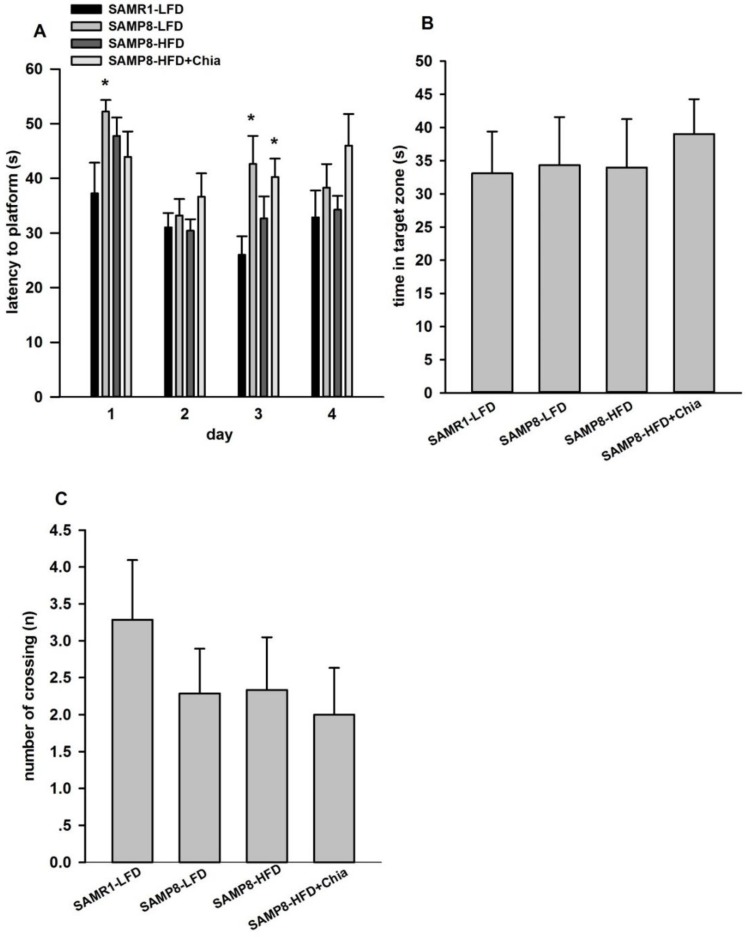
Morris Water Maze (MWM) test of mice from different groups. (**A**) Mean latency in the hidden platform test; (**B**) comparison of the time spent in the target quadrant; and (**C**) the number of crossings over the exact, former location of the platform during the probe test. Data are presented as means + SEM for 8 mice per group. * *p* < 0.05 versus SAMR1-LFD group within the same day in (**A**).

As shown in Figure 3A, during navigation trials, compared to SAMR1-LFD group, SAMP8-LFD group demonstrated longer latency to platform at day 1 and day 3, and SAMP8-HFD+Chia group also showed longer latency to platform at day 3. During the spatial memory testing, there is no difference for time spent in target quadrant and the number of crossings among groups (Figure 3B,C). Collectively, our results suggested that during navigation trials, mice in SAMP8-LFD group demonstrated impaired learning ability compared to SAMR1-LFD group, while HFD utilized in our present model could not worsen the impaired learning ability, and chia seed has no effect on learning and memory ability for HFD fed SAMP8 mice of 28 weeks old.

### 3.4. Protein Expression Related to Aβ Pathology

**Figure 4:**
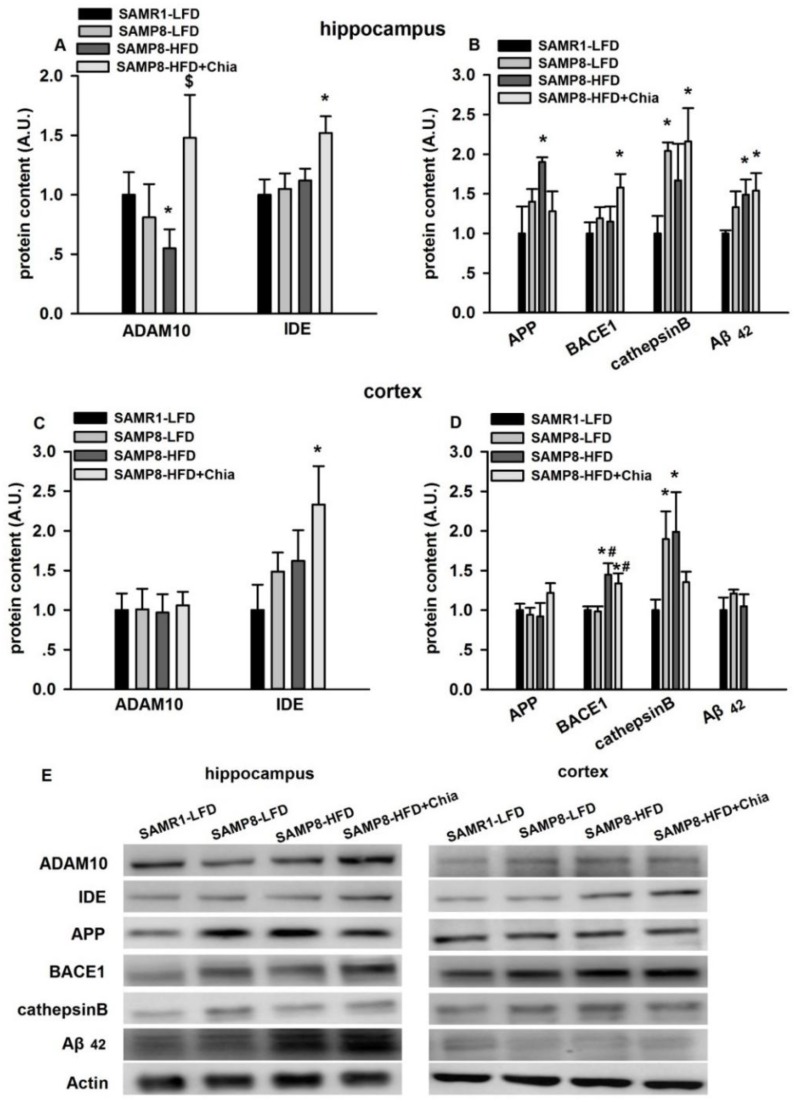
ADAM10, insulin degrading enzyme (IDE), amyloid precursor protein (APP), BACE1, cathepsin B and Aβ_42_ protein expression in the hippocampus and cortex. Protein expression of ADAM10, IDE, APP, BACE1, cathepsin B and Aβ_42_ in the hippocampus (**A**,**B**) and cortex (**C**,**D**) from all groups measured via Western blotting. Representative blots are shown in panel (**E**). A.U. means arbitrary units. Data are presented as means + SEM for 8 mice per group. * *p* < 0.05 versus SAMR1-LFD group. # *p* < 0.05 versus SAMP8-LFDgroup. $ *p* < 0.05 versus P8HF group.

As shown in Figure 4A, the hippocampus, compared to SAMR1-LFD group, SAMP8-HFD group had reduced ADAM10 protein expression, and SAMP8-HFD+Chia group has elevated IDE protein expression; compared to SAMP8-HFD group, SAMP8-HFD+Chia group also had elevated ADAM10 protein expression. Compared to SAMR1-LFD group, SAMP8-LFD group had elevated cathepsin B protein expression; and SAMP8-HFD group had increased APP and Aβ_42_ protein expression; and SAMP8-HFD+Chia group had elevated BACE1, cathepsin B and Aβ_42_ protein expression (Figure 4B). In cortex, compared to SAMR1-LFD group, SAMP8-HFD+Chia group had elevated IDE protein expression (Figure 4C). Compared to SAMR1-LFD group, SAMP8-LFD group had elevated cathepsin B protein expression, SAMP8-HFD group also had elevated BACE1 and cathepsin B protein expression, and SAMP8-HFD+Chia group had elevated BACE1 protein expression. Compared to SAMP8-LFDgroup, SAMP8-HFD and SAMP8-HFD+Chia group also had elevated BACE1 protein expression (Figure 4D). 

### 3.5. Tau Phosphorylation and Tau Kinases Protein Expression

**Figure 5:**
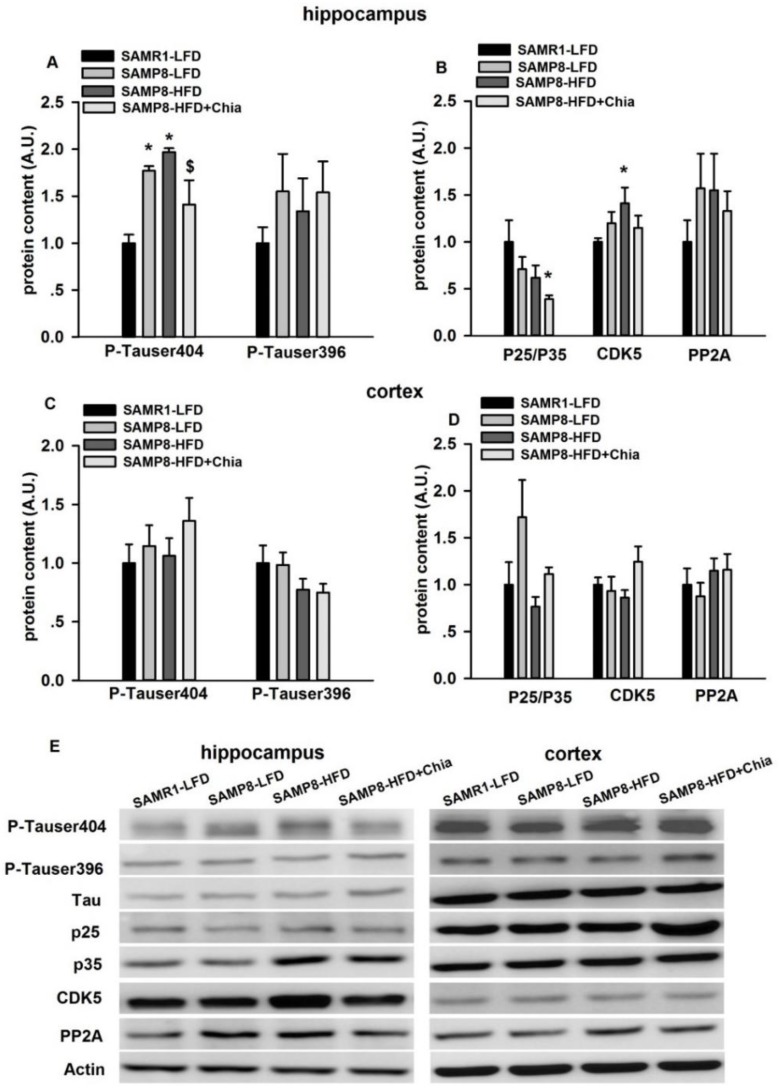
P-Tauserine404&396, p25/35, CDK5 and PP2A protein expression in the hippocampus and cortex. Protein expression of p-Tauserine404&396, p25/35, CDK5 and PP2A in the hippocampus (**A**,**B**) and cortex (**C**,**D**) from all groups measured via Western blotting. Representative blots are shown in panel (**E**). A.U. means arbitrary units. Data are presented as means + SEM for 8 mice per group. * *p* < 0.05 versus SAMR1-LFD group. $ *p* < 0.05 versus SAMP8-HFD group.

As shown in Figure 5A, in the hippocampus, compared to SAMR1-LFD group, there was elevated phosphorylation of tau at serine404 (p-Tauser404) in SAMP8-LFD and SAMP8-HFD group; compared to SAMP8-HFD group, the protein expression of p-Tauser404 was reduced from SAMP8-HFD+Chia group. We also measured key kinases involved in tau phosphorylation (i.e., p25/35 and CDK5) [26,27] and dephosphorylation (i.e., PP2A) [28]. As shown in Figure 5B, compared to SAMR1-LFD mice, the protein expression of CDK5 was significantly increased from SAMP8-HFD group, while p25/p35 was significantly reduced from SAMP8-HFD+Chia group. There was no difference for protein expression of p-Tau at serine396&404, as well p25/35, CDK5 and PP2A protein from cortex among groups (Figure 5C,D). 

### 3.6. GFAP Protein Expression and Immune-Staining for Aβ_42_ + Ibα-1

**Figure 6:**
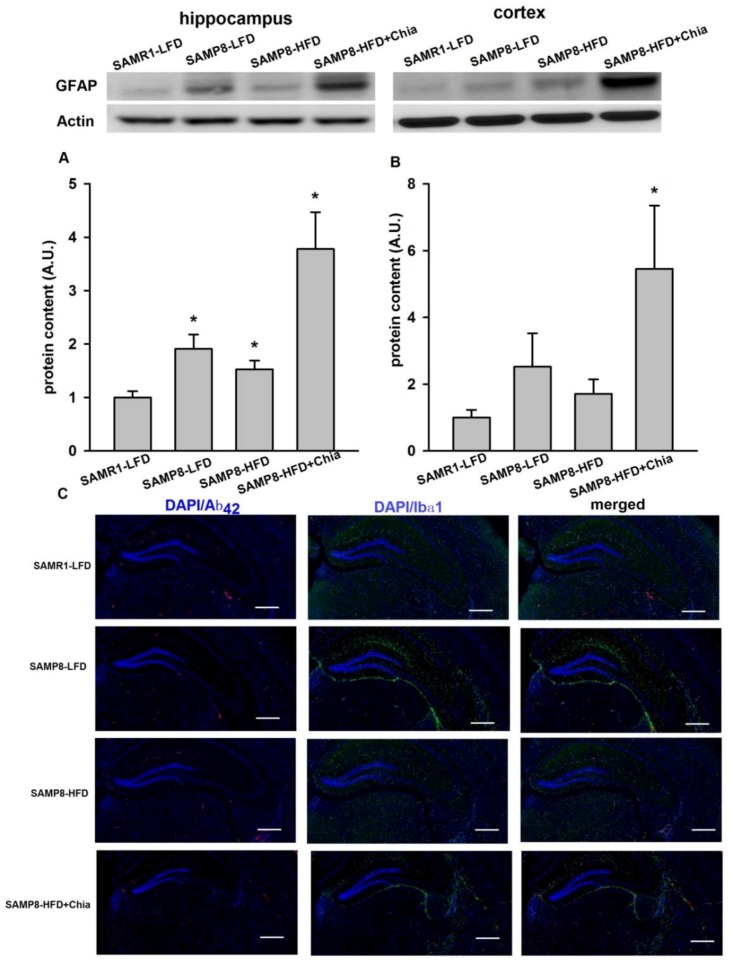
GFAP protein expression in the hippocampus and cortex and immune-staining for Aβ_42_ + Ibα-1. Protein expression of GFAP in the hippocampus (**A**) and cortex (**B**) from all groups measured via Western blotting. Immune-staining for Aβ_42_ + Ibα-1 surrounding hippocampus area measured via immunofluorescence (**C**). Representative blots are shown at the top of quantified data in (**A**,**B**). A.U. means arbitrary units. Data are presented as means + SEM for 8 mice per group in (**A**,**B**). Scale bar 20 μm, magnification ×200 in (**C**). * *p* < 0.05 versus SAMR1-LFD group.

As shown in Figure 6A, in the hippocampus, compared to SAMR1-LFD group, there was elevated protein expression of GFAP from SAMP8-LFD, SAMP8-HFD and SAMP8-HFD+Chia group, this indicates that elevated astrocytes inflammation existed in all SAMP8 mice. In cortex, compared to SAMR1-LFD mice, the protein expression of GFAP was also significantly increased from SAMP8-HFD+Chia group (Figure 6B). We also measured Aβ_42_ + Ibα1 via immunofluorescence, increased Ibα-1 activation was observed from SAMP8-LFD, SAMP8-HFD and SAMP8-HFD+Chia group surrounding the hippocampus area (Figure 6C).

## 4. Discussion

Our study is the very first to explore how chia seed supplementation might improve AD related pathology via HFD fed SAMP8 mice as a model. The main findings of the present study are there was no difference for cognitive function via MWM test for SAMP8 mice fed with LFD and HFD, and chia seed supplementation had no effects on cognitive function in HFD fed SAMP8 mice. As for AD related pathology, chia seed not only increased α-secretase including ADAM10 and IDE, but also increased β-secretase including BACE1 and cathepsin B, with an overall effect of elevation in the hippocampal Aβ_42_ level; chia seed slightly reduced p-Tauser404 in the hippocampus; while an elevation in neuro-inflammation with activation of GFAP and Ibα-1 have been observed post chia seed supplementation.

It has been demonstrated that under HFD condition, SAMP8 mice would demonstrate worsen cognitive impairment via MWM [21] and novel object recognition test (NORT) [29]. However, in our present study, 18 weeks of HFD in SAMP8 mice has no significant effects on cognitive function via MWM test, this is in contrast to the findings by Mehla et al. [21]; while is consistent with the findings by Palomera-Avalos et al. [29]. The inconsistent findings might be related to HFD intervention length, to be specific, the HFD intervention period is 8 weeks in the study by Mehla et al., while 15 weeks in the study by Palomera-Avalos et al.; similarly, our HFD intervention period is 18 weeks. It is likely that a relative short-term HFD feeding (i.e., 8 weeks) might aggravate the non-hippocampal dependent cognitive function via MWM, while this effect might lose with a long-term HFD feeding. It is also likely long-term HFD might worse hippocampal dependent function measured via NORT, instead of MWM. However, owing to equipment limitation, we were unable to measure this. Nevertheless, further studies are required to explore the time-course effects of HFD feeding on cognitive function in SAMP8 mice. To our surprise, chia seed, which contains the richest plant source of ALA [5], demonstrated no beneficial effects on cognitive function in HFD SAMP8 mice. However, it should be realized that chia seed might improve hippocampal dependent function measured via NORT. 

One of the hallmarks of AD is extracellular amyloid plaques consisting of Aβ peptides, generated through the sequential proteolytic cleavage of the amyloid precursor protein (APP) by the β- and γ-secretase [30], which is also known as the amyloidogenic pathway. β-secretases including mainly BACE1 and cathepsin B are the rate-limiting enzyme in Aβ genesis [31]. Insulin degrading enzyme (IDE) is one of the major proteolytic enzymes involved in Aβ degradation [32]. On the other hand, cleavage of APP by α-secretases (i.e., ADAM10) constitutes the non-amyloidgenic pathway, which generates sAPPα and C83, consequently precluding Aβ formation [33]. Therefore, decreased amyloidogenic processing (i.e., reduction in BACE1 and cathepsin B expression), and/or increased non-amyloidgenic processing of APP (i.e., enhanced ADAM10 expression), and/or increased Aβ degradation (elevation in IDE expression) would improve Aβ peptide aggregation. In our present study, HFD resulted in reduction in ADAM10, elevation in APP protein expression in the hippocampus, as well as elevation in BACE1 and cathepsin B protein expression in parietal cortex, which suggests that HFD, *per se*, favors the amyloidogenic pathway of APP, consequently resulted in elevation in Aβ_42_ protein expression, at least in the hippocampus. Chia seed intervention group has elevated ADAM10 and IDE expression in the hippocampus, which suggests chia seed might increase non-amyloidgenic processing of APP and Aβ degradation. However, chia seed group also has increased BACE1, cathepsin B and Aβ_42_ protein expression, indicating an elevation in amyloidogenic pathway. Overall, it is suggested that 10% chia seed intervention with a total of 18 weeks is unable to improve HFD induced Aβ_42_ aggregation in SAMP8 mice, this is consistent with the cognitive function test via MWM.

Another hallmark of AD is neurofibrillary tangles (NFTs) composed of hyperphosphorylated tau protein [34]. Recent evidence now supports that early tau phosphorylation is critical in the development of synaptic dysfunction and neurodegeneration [35]. The phosphorylated tau at serine396&404 has been considered as common markers for the severity of AD [36]. Multiple proline-directed serine-threonine protein kinases including CDK5 are implicated in hyperphosphorylating tau [26], while PP2A is the major tau phosphatase that dephosphorylats tau [28]. The calpain cleavage of p35 will produce p25, which is responsible for CDK5 hyperactivation [27], therefore increased p25/p35 is also associated with hyperphosphorylated tau. In our present study, elevated p-Tau ser404 was observed in the hippocampus from SAMP8 mice under both low fat and high fat diet condition, chia seed could slightly but significantly reduce p-Tau ser404, meanwhile a significant reduction in p25/35 is also observed in the hippocampus from SAMP8-HFD+Chia group. However, no alterations in p-Tauser396/404 nor p25/35, CDK5, PP2A protein expression were observed in cortex. Overall, it is suggested that chia seed supplementation could reduce elevated p-Tau at serine404 in the hippocampus, this might be owing to reduction in p25/35, however, this effect is not able to translate into improvement in learning and memory abilities, at least via MWM.

Neuro-inflammation, which is mediated mainly by activated astrocytes and microglia, also play critical roles in the pathogenesis of AD [37]. Positron emission tomography (PET) analysis suggested that microglia cells were activated in the early stage of AD, even prior to Aβ deposition [38]. Increased GFAP and Ibα-1 protein expression are usually considered as markers for reactive astrogliosis [39] and microglia activation [40], respectively. In our present study, activation of GFAP was observed in the hippocampus from SAMP8 mice under both low fat and high fat diet condition, chia seed group also has elevated GFAP not only in the hippocampus, but also in cortex. Meanwhile, elevated Ibα-1 protein expression via IF was observed in SAMP8-LFD, SAMP8-HFD and SAMP8-HFD+Chia group. We are as yet unable to explain why chia seed induced neuro-inflammation. Previously, Nieman et al. [41] reported that ingestion of 25 g/day milled chia seed for 10 weeks has no influence on inflammation in overweight women, although significantly increased plasma ALA and EPA. In contrast, Vuksan et al. [14] demonstrated that 12 weeks supplementation of Salba in individuals with type 2 diabetes reduced plasma high sensitivity C-reactive protein, suggesting chia seed might exert anti-inflammatory effects. Evidence from animal studies is still lacking, in regards to how chia seed supplementation influences inflammatory processes in different organs, especially in the brain. Nevertheless, our study suggested that chia seed might worse the neuro-inflammation. 

Chia seed, a food native from southern Mexico and northern Guatemala [4] have drawn scientists’ and nutritionist’s attention over the past two decades. Multiple beneficial effects especially in peripheral tissues such as improving glucose tolerance and insulin resistance [6,7,8,10], adiposity [11] and disordered lipid profiles [9,11] have been reported. However, our present study demonstrated that 18 weeks of 10% chia seed supplementation in SAMP8 mice under HFD condition has no influence on learning and memory abilities via MWM. Further animal studies with different dosages and intervention duration of chia seed are required to confirm our present study, as well as other cognitive function test such as NORT. Considering that commercially available chia seed already existed, and that evidences suggest that chia seed might be beneficial for cardiovascular health clinically, our study highlights the significance of comprehensively evaluating effects of chia seed in human body before it can be broadly utilized as a nutritional supplement for regulating metabolic syndrome associated disorder.

Our study has limitations. First of all, there is no control group for SAMR1 mice fed with high fat diet, therefore we are unable to confirm whether the high fat resistance of this mouse strain was specific to SAMP8 mice. Secondly, we only utilized SAMP8 mice as an AD model, therefore the extrapolation of our results to other AD models are limited. It is likely that chia seed might be beneficial for improving AD related pathology in other animal models, and in AD subjects. Further studies are needed to explore how chia seed supplementation will affect AD pathology with different animal models and subjects with cognitive impairment.

## 5. Conclusions

In conclusion, we demonstrate that chia seed supplementation has no effects on cognitive function via MWM in HFD fed SAMP8 mice. This might be associated with the fact that chia seed supplementation not only increased key enzymes involved in non-amyloidogenic pathways and reduced p-Tauser404, but also increased key enzymes involved in amyloidogenic pathways and neuro-inflammation. Our study might be of importance to the application of chia seed in human population.

## Figures and Tables

**Table 1:** Fatty acid profile of chia seed.

Fatty Acid Composition	g/100 g of Total Fatty Acid Content (*N* = 3)
C14:0	2.06 ± 0.38
C16:0	2.06 ± 0.40
C18:0	0.95 ± 0.22
C18:1 *n*-9	1.9 ± 0.14
C18:2 *n*-6	5.55 ± 0.37
C18:3 *n*-3	19.5 ± 0.26
C20:0	0.08 ± 0.0
Total *n*-3 PUFA	19.6 ± 0.52
Total *n*-6 PUFA	5.78 ± 0.24
Total *n*-9 PUFA	1.95 ± 0.25
Total SFA	3.18 ± 0.16
Total MUFA	2.02 ± 0.44
Total PUFA	25.2 ± 0.37

Data is shown a mean ± SEM. MUFA, monounsaturated fatty acid; PUFA, polyunsaturated fatty acid; SFA, saturated fatty acid.

## References

[1] Letra L., Santana R., Seica I. (2014). Obesity as a risk factor for Alzheimer’s disease: The role of adipocytokines. Metab. Brain Dis..

[2] De la Monte S.M. (2014). Relationships between diabetes and cognitive impairment. Endocrinol. Metab. Clin. N. Am..

[3] Martins I.J., Hone E., Foster J.K., Sunram-Lea S.I., Gnjec A., Fuller S.J., Nolan D., Gandy S.E., Martins R.N. (2006). Apolipoprotein E, cholesterol metabolism, diabetes, and the convergence of risk factors for Alzheimer’s disease and cardiovascular disease. Mol. Psychiatry.

[4] Muñoz Loreto A., Cobos A. (2013). Olga DiazJosé Miguel Aguilera Chia Seed (*Salvia hispanica*): An Ancient Grain and a New Functional Food. Food Rev. Int..

[5] Coates W. (2011). Protein content, oil content and fatty acid profiles as potential criteria to determine the origin of commercially grown chia (*Salvia hispanica* L.). Ind. Crops Prod..

[6] Vuksan V., Jenkins A.L., Brissette C., Choleva L., Jovanovski E., Gibbs A.L., Bazinet R.P., Au-Yeung F., Zurbau A., Ho H.V. (2017). Salba-chia (*Salvia hispanica* L.) in the treatment of overweight and obese patients with type 2 diabetes: A double-blind randomized controlled trial. Nutr. Metab. Cardiovasc. Dis..

[7] Vuksan V., Jenkins A.L., Dias A.G., Lee A.S., Jovanovski E., Rogovik A.L., Hanna A. (2010). Reduction in postprandial glucose excursion and prolongation of satiety: Possible explanation of the long-term effects of whole grain Salba (*Salvia hispanica* L.). Eur. J. Clin. Nutr..

[8] Ho H., Lee A.S., Jovanovski E., Jenkins A.L., Desouza V., Vuksan R. (2013). Effect of whole and ground Salba seeds (*Salvia hispanica* L.) on postprandial glycemia in healthy volunteers: A randomized controlled, dose-response trial. Eur. J. Clin. Nutr..

[9] Guevara-Cruz M., Tovar A.R., Aguilar-Salinas C.A., Medina-Vera I., Gil-Zenteno L., Hernandez-Viveros I., Lopez-Romero P., Ordaz-Nava G., Canizales-Quinteros S., Guillen Pineda L.E. (2012). A dietary pattern including nopal, chia seed, soy protein, and oat reduces serum triglycerides and glucose intolerance in patients with metabolic syndrome. J. Nutr..

[10] Marineli Rda S., Moura C.S., Moraes E.A., Lenquiste S.A., Lollo P.C., Morato P.N., Amaya-Farfan J., Marostica M.R. (2015). Chia (*Salvia hispanica* L.) enhances HSP, PGC-1alpha expressions and improves glucose tolerance in diet-induced obese rats. Nutrition.

[11] Chicco A.G., D’Alessandro M.E., Hein G.J., Oliva M.E., Lombardo Y.B. (2009). Dietary chia seed (*Salvia hispanica* L.) rich in alpha-linolenic acid improves adiposity and normalises hypertriacylglycerolaemia and insulin resistance in dyslipaemic rats. Br. J. Nutr..

[12] Poudyal H., Panchal S.K., Waanders J., Ward L., Brown L. (2012). Lipid redistribution by alpha-linolenic acid-rich chia seed inhibits stearoyl-CoA desaturase-1 and induces cardiac and hepatic protection in diet-induced obese rats. J. Nutr. Biochem..

[13] Jin F., Nieman D.C., Sha W., Xie G., Qiu Y., Jia W. (2012). Supplementation of milled chia seeds increases plasma ALA and EPA in postmenopausal women. Plant. Foods Hum. Nutr..

[14] Vuksan V., Whitham D., Sievenpiper J.L., Jenkins A.L., Rogovik A.L., Bazinet R.P., Vidgen E., Hanna A. (2007). Supplementation of conventional therapy with the novel grain Salba (*Salvia hispanica* L.) improves major and emerging cardiovascular risk factors in type 2 diabetes: Results of a randomized controlled trial. Diabetes Care.

[15] Nieman D.C., Cayea E.J., Austin M.D., Henson D.A., McAnulty S.R., Jin F. (2009). Chia seed does not promote weight loss or alter disease risk factors in overweight adults. Nutr. Res..

[16] Gao H., Yan P., Zhang S., Nie S., Huang F., Han H., Deng Q., Huang Q., Yang W., Wu H. (2016). Chronic alpha-linolenic acid treatment alleviates age-associated neuropathology: Roles of PERK/eIF2alpha signaling pathway. Brain Behav. Immun..

[17] Lee A.Y., Choi J.M., Lee J., Lee M.H., Lee S., Cho E.J. (2016). Effects of Vegetable Oils with Different Fatty Acid Compositions on Cognition and Memory Ability in Abeta25-35-Induced Alzheimer’s Disease Mouse Model. J. Med. Food.

[18] Morley J.E., Farr S.A., Flood J.F. (2002). Antibody to amyloid beta protein alleviates impaired acquisition, retention, and memory processing in SAMP8 mice. Neurobiol. Learn. Mem..

[19] Canudas A.M., Gutierrez-Cuesta J., Rodriguez M.I., Acuna-Castroviejo D., Sureda F.X., Camins A., Pallas M. (2005). Hyperphosphorylation of microtubule-associated protein tau in senescence-accelerated mouse (SAM). Mech. Ageing Dev..

[20] Nomura Y.Y. (1999). Okuma Age-related defects in lifespan and learning ability in SAMP8 mice. Neurobiol. Aging.

[21] Mehla J., Chauhan B.C., Chauhan N.B. (2014). Experimental induction of type 2 diabetes in aging-accelerated mice triggered Alzheimer-like pathology and memory deficits. J. Alzheimers Dis..

[22] Morris R.G. (1990). Toward a representational hypothesis of the role of hippocampal synaptic plasticity in spatial and other forms of learning. Cold Spring Harb. Symp. Quant. Biol..

[23] Wan Z., Thrush A.B., Legare M., Frier B.C., Sutherland L.N., Williams D.B., Wright D.C. (2010). Epinephrine-mediated regulation of PDK4 mRNA in rat adipose tissue. Am. J. Physiol. Cell. Physiol..

[24] Chen N., Zhou L., Zhang Z., Xu J., Wan Z., Qin L. (2014). Resistin induces lipolysis and suppresses adiponectin secretion in cultured human visceral adipose tissue. Regul. Pept..

[25] Cheng J., Chen L., Han S., Qin L., Chen N. (2015). Zhongxiao Wan Treadmill running and rutin reverse high fat diet induced cognitive impairment in diet induced obese mice. J. Nutr. Health Aging.

[26] Metcalfe M.J.M., Figueiredo-Pereira E. (2010). Relationship between tau pathology and neuroinflammation in Alzheimer's disease. Mt. Sin. J. Med..

[27] Patrick G.N., Zukerberg L., Nikolic M., de la Monte S., Dikkes P., Tsai L.H. (1999). Conversion of p35 to p25 deregulates Cdk5 activity and promotes neurodegeneration. Nature.

[28] Liu F., Grundke-Iqbal I., Iqbal K., Gong C.X. (2005). Contributions of protein phosphatases PP1, PP2A, PP2B and PP5 to the regulation of tau phosphorylation. Eur. J. Neurosci..

[29] Palomera-Avalos V., Grinan-Ferre C., Puigoriol-Ilamola D., Camins A., Sanfeliu C., Canudas A.M., Pallas M. (2017). Resveratrol Protects SAMP8 Brain under Metabolic Stress: Focus on Mitochondrial Function and Wnt Pathway. Mol. Neurobiol..

[30] Herrup K. (2010). Reimagining Alzheimer;s disease—An age-based hypothesis. J. Neurosci..

[31] Hook V., Schechter I., Demuth H.U., Hook G. (2008). Alternative pathways for production of beta-amyloid peptides of Alzheimer's disease. Biol. Chem..

[32] Llovera R.E., de Tullio M., Alonso L.G., Leissring M.A., Kaufman S.B., Roher A.E., Gay G.D., Morelli L., Castano E.M. (2008). The catalytic domain of insulin-degrading enzyme forms a denaturant-resistant complex with amyloid beta peptide: Implications for Alzheimer disease pathogenesis. J. Biol. Chem..

[33] Kang J., Lemaire H.G., Unterbeck A., Salbaum J.M., Masters C.L., Grzeschik K.H., Multhaup G., Beyreuther K., Muller-Hill B. (1987). The precursor of Alzheimer’s disease amyloid A4 protein resembles a cell-surface receptor. Nature.

[34] Guo J.L.V., Lee M. (2011). Seeding of normal Tau by pathological Tau conformers drives pathogenesis of Alzheimer-like tangles. J. Biol. Chem..

[35] Hochgrafe K., Sydow A., Mandelkow E.M. (2013). Regulatable transgenic mouse models of Alzheimer disease: Onset, reversibility and spreading of Tau pathology. FEBS J..

[36] Hu Y.Y., He S.S., Wang X., Duan Q.H., Grundke-Iqbal I., Iqbal K., Wang J. (2002). Levels of nonphosphorylated and phosphorylated tau in cerebrospinal fluid of Alzheimer’s disease patients: An ultrasensitive bienzyme-substrate-recycle enzyme-linked immunosorbent assay. Am. J. Pathol..

[37] Rogers J., Webster S., Lue L.F., Brachova L., Civin W.H., Emmerling M., Shivers B., Walker D., McGeer P. (1996). Inflammation and Alzheimer’s disease pathogenesis. Neurobiol. Aging.

[38] Yasuno F., Kosaka J., Ota M., Higuchi M., Ito H., Fujimura Y., Nozaki S., Takahashi S., Mizukami K., Asada T. (2012). Increased binding of peripheral benzodiazepine receptor in mild cognitive impairment-dementia converters measured by positron emission tomography with [(1)(1)C]DAA1106. Psychiatry Res..

[39] Simpson J.E., Ince P.G., Lace G., Forster G., Shaw P.J., Matthews F., Savva G., Brayne C., Wharton S.B. (2010). Astrocyte phenotype in relation to Alzheimer-type pathology in the ageing brain. Neurobiol. Aging.

[40] Pomilio C., Pavia P., Gorojod R.M., Vinuesa A., Alaimo A., Galvan V., Kotler M.L., Beauquis J., Saravia F. (2016). Glial alterations from early to late stages in a model of Alzheimer’s disease: Evidence of autophagy involvement in Abeta internalization. Hippocampus.

[41] Nieman D.C., Gillitt N., Jin F., Henson D.A., Kennerly K., Shanely R.A., Ore B., Su M., Schwartz S. (2012). Chia seed supplementation and disease risk factors in overweight women: A metabolomics investigation. J. Altern. Complement. Med..

